# Enhanced Hole Injection in AlGaN-Based Ga-Polar Ultraviolet Light-Emitting Diodes with Polarized Electric-Field Reservoir Electron Barrier

**DOI:** 10.3390/mi15060762

**Published:** 2024-06-06

**Authors:** Zhuang Zhao, Yang Liu, Peixian Li, Xiaowei Zhou, Bo Yang, Yingru Xiang

**Affiliations:** 1 School of Advanced Materials and Nanotechnology, Xidian University, Xi’an 710071, China; 22141214314@stu.xidian.edu.cn (Z.Z.); yliu_333@stu.xidian.edu.cn (Y.L.); yangbo1@stu.xidian.edu.cn (B.Y.) 22141214236@stu.xidian.edu.cn (Y.X.); 2 State Key Discipline Laboratory of Wide Band Gap Semiconductor Technology, Xidian University, Xi’an 710071, China

**Keywords:** ultraviolet light-emitting diodes, electron blocking layer, APSYS

## Abstract

In this study, we propose a polarized electron blocking layer (EBL) structure using Al_x_Ga_1−x_N/Al_x_Ga_1−x_N to enhance the internal quantum efficiency (IQE) of AlGaN-based ultraviolet light-emitting diodes (UV LEDs). Our findings indicate that this polarized EBL structure significantly improves IQE compared to conventional EBLs. Additionally, we introduce an electric-field reservoir (EFR) optimization method to maximize IQE. Specifically, optimizing the polarized EBL structure of Al_x_Ga_1−x_N/Al_x_Ga_1−x_N enhances the hole drift rate, resulting in an IQE improvement of 19% and an optical output power increase of 186 mW at a current of 210 mA.

## 1. Introduction

In the last few years, AlGaN-based UV light-emitting diodes have begun to possess several advantages, including compact size, low power consumption, tunable wavelength, eco-friendliness, and extended lifespan. These attributes have led to their widespread adoption across various sectors, including sterilization and disinfection, sensing, water purification, medical applications, and non-line-of-sight communication [1]. However, despite the ongoing advancements in material and device preparation technologies, AlGaN-based deep-ultraviolet LEDs face challenges compared to the established InGaN-based visible LEDs. These challenges include the weak confinement of multiple quantum wells (MQWs), low p-doping efficiency of high-Al composition AlGaN, and the quantum-confinement Stark effect (QCSE) induced by polarization fields [2,3], which means that UV LED development still faces great challenges such as severe electron leakage, poor hole injection, a low radiative compounding rate, and especially low radiated power due to significantly low external quantum efficiency (external quantum efficiency, EQE) [4,5]. Therefore, the investigation of methods to enhance UV LED efficiency holds significant importance across various application domains. The external quantum efficiency of AlGaN-based UV LEDs can be enhanced through improvements in internal quantum efficiency (IQE) and light extraction efficiency (LEE) [6]. IQE relies on the effective injection of carriers into the active region and the generation of radiative recombination within that region. Internal quantum efficiency = (number of photons spilled into the air/total number of photons produced by the PN junction) and its enhancement is mainly related to the setting of the epitaxial material, which is determined by the dynamic parameters of its heterojunction, which include the following: 1. Dynamic parameter of the thickness of the heterojunction: there is an optimal range of the thickness of the structure in each layer, and too thick as well as too low cannot ensure the non-uniform distribution of the carriers in each trap. 2. Dynamic range of the Al doping concentration: with too high a concentration of Al, the carrier lifetime is shortened and the radiation compounding rate increases, but its high doping concentration can introduce defects. 3. Range: the high doping concentration of Al reduces the carrier lifetime and increases the radiative composite rate, but its high doping concentration introduces defects. 4. Lattice matching factor of heterojunction: a decrease in its dislocation density increases the nonradiative composite rate of the heterojunction by less [7].

As the Al composition in AlGaN increases from 0% to 100%, the activation energy of Mg rises from 170 meV to 470 meV [8]. This notable increase in activation energy leads to the activation of only a few Mg dopants for hole generation in AlGaN materials with high Al composition at room temperature [9]. Consequently, the p-type doping efficiency of AlGaN materials diminishes with increasing Al composition. This reduced efficiency results in inadequate hole generation, exacerbating the already pronounced imbalance between electron and hole injection. Additionally, weak electron confinement contributes to severe electron leakage [10]. The nonradiative recombination of leakage electrons with holes in the p-type region serves as the primary cause of insufficient hole injection, resulting in a weak radiative recombination rate and, consequently, low output power. Cho et al. showcased that electron leakage induces the generation of an electric field in the p-type layer, subsequently contributing to efficiency degradation [11]. Additionally, Chu et al. constructed UV LEDs and examined the correlation between efficiency degradation, electron leakage, and the Auger–Meitner effect [12]. Their findings elucidated that electron leakage predominantly triggers efficiency degradation. Ren et al. demonstrated that the severe leakage current and efficiency degradation of UV LEDs at high injection currents are primarily attributed to substantial electron leakage and inadequate hole injection [13]. Hence, the paramount approach to optimizing UV LED performance lies in reducing electron leakage and enhancing hole injection. To address these challenges and improve the internal quantum efficiency of LEDs, electron blocking layer (EBL) structures have been proposed and widely adopted. By integrating an EBL between the active region and the p-type region, carrier injection is enhanced, consequently boosting internal quantum efficiency. Typically positioned between MQWs and the p-type layer, the EBL consists of AlGaN material with a wider bandgap than that of the p-type layer. This AlGaN layer forms a potential barrier ahead of the p-type layer, effectively blocking electron leakage and enhancing the luminous efficiency of AlGaN-based LEDs [14].

In addition, most UVLEDs are grown in heterojunction structures on sapphire substrates in the (0001) c-plane by MOCVD [15,16], and although sapphire substrates provide low-cost templates for UVLEDs, the larger 14% lattice mismatch between sapphire and AlN leads to an increase in the number of significant non-helical dislocations and point defects, and helical dislocations and point defects in AlGaN are a major contributor to nonradiative composites of UVLEDs [17]; in order to overcome these limitations, a variety of methods to form AlN templates with low defect densities on sapphire substrates have been explored. They include migration-enhanced metal–organic chemical vapor deposition [18], the migration-enhanced lateral epitaxial overgrowth of AlN [19], etc. Therefore, we used AlN as the buffer layer of choice this time.

Numerous researchers have dedicated their efforts and made significant contributions to this field. Hirayama et al. (2010) introduced the utilization of multiple quantum barrier (MQB) EBLs. Through comparative analysis with conventional single-layer EBL structures, they demonstrated that the enhanced EBL effectively suppresses electron leakage. The results underscored the efficacy of the superlattice EBL in reducing electron leakage by leveraging the multiple reflection effect on the electron wave function [20]. Zheng et al. fabricated a multidimensional Mg-doped superlattice in Al-rich AlGaN to elevate the hole concentration, a pivotal aspect in the design of AlGaN-based UV LEDs [21]. Zhang et al. synthesized superlattice p-type EBLs to minimize electron leakage and boost hole injection, thus mitigating efficiency degradation [22]. Their findings indicated that augmenting hole injection in multiple quantum wells aids in mitigating electron leakage by more effectively utilizing electrons through radiative recombination. In efforts to enhance the hole injection efficiency, Kim et al. endeavored to alter the material of the final layer of the quantum barrier within the active region to InAlGaN. By introducing a reasonable component, they established a bilayer InAlGaN/AlGaN structure in conjunction with the original EBL, thereby enhancing hole transport [23]. Furthermore, Vurgaftman and Chuang et al. introduced a gradient EBL structure, wherein a gradient hole reservoir is created by varying the Al component in the EBL, thereby effectively controlling the electron leakage barrier [24,25]. Building upon this concept, Yi et al. proposed a gradient superlattice EBL structure, where the gradient EBL exhibits periodic alterations, leading to a reduction in polarization charge and lattice mismatch [26]. Zhang et al. enhanced the hole drift velocity by elevating the kinetic energy of the holes within the p-type region. They achieved this by minimizing the energy difference (ΔΦ = Φ_p-EBL_ − Ek_holes_) between the kinetic energy of holes (Ek_holes_) and the height of the valence band barrier of the p-ELB (Φ_p-ELB_), thus facilitating the thermionic emission of holes across the p-ELB. Additionally, they increased the doping of the Mg concentration to augment hole mobility, consequently boosting the kinetic energy of the holes [27]. However, due to the low doping efficiency of p-type layer group III nitride Mg, there is limited room to further increase the hole concentration. Therefore, Zhang et al. suggested employing a polarization-induced electric field within a hole gas pedal to elevate the drift velocity of the holes [28]. Building on the insights gained from the aforementioned studies, it is proposed to utilize AlGaN/AlGaN as a polarized EBL. Moreover, the incorporation of the EFR within the polarized EBL is suggested to enhance hole injection efficiency and improve electron confinement capability.

In this study, UV LEDs featuring a polarized EBL with EFR, emitting at a peak wavelength of 365 nm, were investigated. Simulation results were obtained by comparing their photovoltaic performance with that of conventional LEDs, as well as LEDs lacking a polarized EBL with an EFR layer, in terms of optical output power, current voltage (I-V) characteristics, and internal quantum efficiency (IQE). The findings reveal that the polarized EBL layer structure, optimized with Al_x_Ga_1−x_N/Al_x_Ga_1−x_N/ Al_x_Ga_1−x_N, exhibits higher IQE and optical output power. This is attributed to its unique barrier structure, which induces a local electric field at the barrier interface, enhancing hot-ion emission and in-band tunneling, thereby effectively promoting hole injection. This enhancement is expected to maximize improvements in the carrier injection process and internal quantum efficiency.

## 2. Materials and Methods

**Figure 1:**
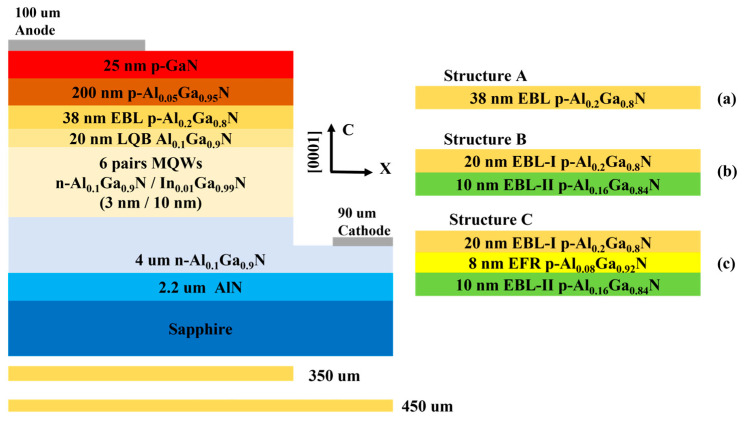
Comparison of AlGaN-based UV-LEDs with different structures, (a) conventional p-EBL structure A, (b) polarization p-EBL structure B, (c) polarization p-EBL and EFR structure C.

The 2D AlGaN/InGaN UV LED structure constructed using APSYS (2016 version) simulation is shown in Figure 1; structure A is the UV LED structure model of a conventional EBL, structure B is the UV LED structure model of a polarized EBL, and structure C is the UV LED structure model of a polarized EBL with the EFR layer. The three models are identical except for the different EBL. The structures of the AlGaN UV LEDs are, in order from bottom to top, as follows: c-plane sapphire substrate, AlN buffer layer with a thickness of 2.2 μm, n-Al_0.1_Ga_0.9_N layer with a thickness of 4 μm (n = 5 × 10^18^ cm^−3^), and 6-periodic n-Al_0.1_Ga_0.9_N/In_0.01_Ga_0.99_N multi-quantum wells (MQWs) (n = 3 × 10^17^ cm^−3^) with potential well and barrier thicknesses of 3 nm and 10 nm, respectively. The LQB layer of Al_0.1_Ga_0.9_N has a thickness of 20 nm, the p-Al_0.2_Ga_0.8_N conventional EBL has a thickness of 38 nm (p = 5 × 10^18^ cm^−3^), the p-Al_0.05_Ga_0.95_N layer has a thickness of 200 nm (p = 5 × 10 ^18^cm^−3^), and the p-GaN layer has a thickness of 25 nm (p = 1 × 10^20^ cm^−3^). The polarized EBLs for structure B were EBL-I with a thickness of 20 nm p-Al_0.2_Ga_0.8_N and EBL-II with a thickness of 10 nm p-Al_0.16_Ga_0.84_N (p = 5 × 10^18^ cm^−3^). For the polarized EBL with an EFR layer of structure C, the EBL-I of p-Al_0.2_Ga_0.8_N has a thickness of 20 nm, the EFR layer of p-Al_0.08_Ga_0.92_N has a thickness of 8 nm, and the EBL-II of p-Al_0.16_Ga_0.84_N has a thickness of 10 nm (p = 5 × 10^18^ cm^−3^). Si and Mg were used as n-type and p-type doping sources, respectively. The electrode structure of the LEDs was a transverse electrode structure with an ohmic contact between the positive electrode and the p-GaN contact layer, and the table size was set to 450 × 450 μm^2^.

In the quest to ascertain the fundamental factors driving carrier transport, we conducted numerical calculations employing finite element analysis. These calculations were executed by utilizing the commercial software APSYS (2016 version), renowned for its capability to compute the valence bands of strain-fibrillated zincite nitrides through a 6 × 6 k·p mode [25]. During the numerical simulations, the ionization energy of the p dopant was 220 meV for GaN and 470 meV for AlN [29,30]. The ionization energy increases linearly with the Al content of the AlxGa_1−x_N alloy. The carrier transport is also sensitive to the energy band offset ratio, which is set to be 65/35 for AlGaN/AlGaN heterojunctions [30]. Based on methods developed by Fiorentini et al., polarization charges are calculated [31]. Since polarization charge can be screened by defect and dislocations, 50% of the theoretical polarization charges are considered in the device simulation [32].

Moreover, the Shockley–Read–Hall (SRH) recombination lifetime and Auger–Meitner effect coefficient are set to be 20 ns and 1 × 10^−46^ cm^6^/s for the nonradiative recombination in MQWs, respectively. The I-V characteristic is influenced by the polarization charge screening coefficient, and the SRH lifetime as well as Auger–Meitner effect settings contribute to the light output power–current (L-I) characteristics. The other material parameters of nitrides adopted in the simulation can be found elsewhere. The other material parameters of nitrides adopted in the simulation can be found elsewhere [33]. Also, the tunneling process for holes and electrons is considered in our calculation by employing the transfer matrix method and the one-dimensional Schrödinger equation.

## 3. Results

**Figure 2:**
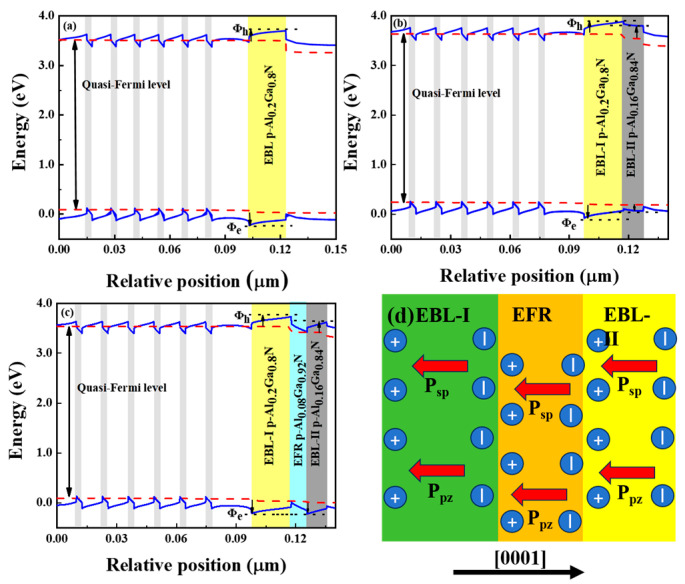
Energy band diagrams of (**a**) structure A, (**b**) structure B, and (**c**) structure C. The data are all calculated at 210 mA. (**d**) Schematic of Ga polar charge distribution due to spontaneous and piezoelectric polarization at the interface of Al_x_Ga_1−x_N and Al_x_Ga_1−x_N.

To illustrate the potential mechanisms of performance improvement, we plotted the energy band diagrams of the three devices and explained them in detail. Figure 2a–c show the energy band diagrams (blue line) and quasi-Fermi energy levels (red dotted dashed line) of devices A, B, and C, respectively, at an injection current of 210 mA. For the three-group structure, the positively polarized charge is formed at the last quantum barrier (LQB)/EBL interface in the presence of polarization. Thus, the conduction band of the LQB is significantly pulled down to form a valence band barrier that will block the injection of holes into the MQW. In order to reveal the ability of electrons to block and holes to inject into the potentials, we define the potential difference between the conduction band and the quasi-Fermi energy level as the height of the electron and hole potentials, denoted by Φe and Φh, respectively. The values of the electron and hole barriers for the three sets of structures are illustrated in Table 1.

In the conventional p-EBL, the energy band shift due to the presence of LQB positively polarized charge leads to a high valence band barrier for hole injection as well as a low conduction band barrier for electron leakage, whereas in structures B and C, the polarization of the EBL-II and EFR layers reduces the amount of positively polarized charge-induced energy band shift, which lowers the valence band barrier and raises the conduction band barrier in favor of hole injection as well as blocking the electron leakage. The order of the valence band barriers of the EBL is as follows: structure B > structure A > structure C, and the order of the conduction band barriers of the EBL is as follows: structure B > structure C > structure A. Compared with structure A, polarized p-EBL structure B and polarized p-EBL and EFR structure C not only block the diffusion of electrons into the p-region but also improve the hole injection efficiency. And under the effect of spontaneous polarization and piezoelectric polarization, the EBL energy band is tilted, and the slope of the energy band tilt is equal to the average electric field strength. For the triangular potential barriers, the relationship between the average slope of the energy band tilt ($\bar{S}$) and the average forbidden band width (${\bar{E}}_{g}$) as well as the EBL width (*w*) is shown in Equation (1):

(1) $$\bar{S}={\bar{E}}_{g}/\left(q\cdot w\right),$$

where ($\bar{S}$) is the average slope of the energy band tilt, (${\bar{E}}_{g}$) is the average forbidden band width, and (*w*) is the heterojunction width. The slope of the energy band tilt in the EBL is dictated by the electric field within the EBL, serving as an indicator of the potential barrier for carrier transport within the EBL. A higher electric field in the EBL corresponds to a larger slope, indicating a higher barrier and greater difficulty for carriers to traverse the EBL. The average forbidden bandwidths in the EBL are arranged as follows: structure A > structure B > structure C. The ordering of the slopes of the energy band tilts in the EBL is as follows: structure A > structure B > structure C. This implies that the polarized p-EBL and the EFR structure C exhibit the lowest energy band slopes in the EBL compared to structures A and B, facilitating the transport of holes within the EBL. For structure C, the tunneling effect occurs in the EFR layer in the middle of the double electron barrier layer under the condition of increasing current injection throughout. This is mainly attributed to the polarization field effect generated at the interface of the EBL-I, EFR layer, and EBL-II (between the EFR and the quantum barrier) as in Figure 2d, that is, the polarization of the electric field of p-EBL-I and the polarization of the quantum barrier of p-EBL-II minus the polarization of the EFR is shown in Equation (2):

(2) $$\Delta p=p^{QB}-p^{EFR},$$

where p^QB^ is the EBL layer polarization intensity and p^EFR^ is the EFR layer polarization intensity. Due to the presence of the polarization effect, a negatively polarized charge is generated at the interface, which then attracts holes, and since the hole concentration in the EBL-I layer is higher than that in the EFR layer, there is hole accumulation in the EFR layer portion, and a slight hole depletion layer occurs in the EBL-I layer at the same time, generating a local electric field, as in Figure 2d. The direction of the electric field is opposite to [0001], which can well increase the kinetic energy of the incident nonequilibrium holes, and the electric field at the heterojunction interface will not be shielded by free carriers. Assuming that the holes are distributed according to the Fermi–Dirac distribution function *P*(*E*), denoting the state of valence band densities in the hole donor, the probability of finding a hole in the last quantum potential is shown in Equation (3):

(3) $$Ph=\int_{E\geq \left\{0,\left(\Phi_{EBL}-E_{k}^{hole}\right)\right\}}^{+\infty }F\left(E\right)\cdot \frac{P\left(E\right)dE}{\int_{0}^{+\infty }F\left(E\right)\cdot P\left(E\right)dE}$$

The concentration (p) of these holes crossing the p-EBL can be expressed as p = P·Ph, where p represents the nonequilibrium hole concentration in the hole layer. Thus, p can be affected by both P and Ph. When Φ*_EBL_* is small enough, the drift rate of the holes is large enough to promote hole injection.

**Figure 3:**
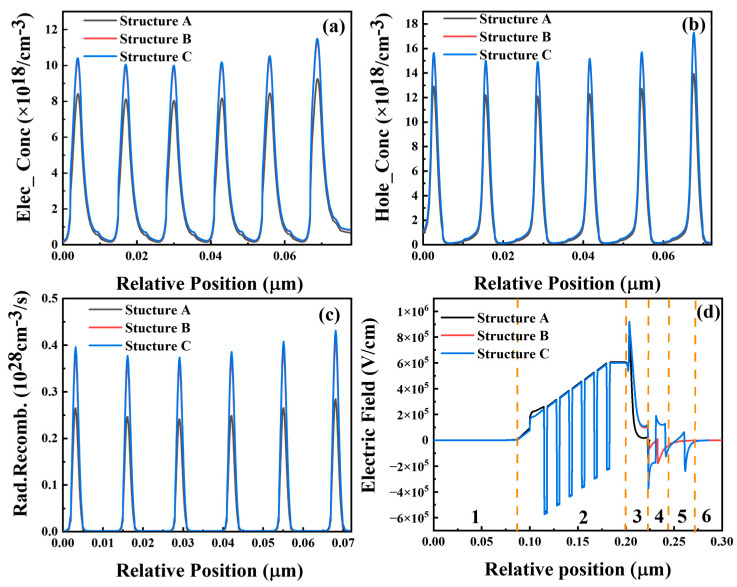
(**a**) Electron concentration and (**b**) hole concentration in MQW region at an injection current density of 210 mA of structures A, B, and C. (**c**) Radiative recombination rates; (**d**) equilibrium electric field distributions of AlGaN-based UV LEDs with structures A, B, and C.

To elucidate the enhanced performance of structures B and C, Figure 3a–c illustrate the electron and hole distribution profiles within the active region, along with the carrier complexation rate profiles. The augmentation of hole and electron concentrations in structures B and C signifies the improved carrier confinement capability and hole injection efficiency of the polarized EBL structure. Additionally, the carrier complexation rates distinctly indicate the enhancement of carrier complexation within the active region as the carrier concentration increases.

To illustrate the localized electric field introduced by the EFR layer, we plotted the equilibrium electric field distributions of the LEDs for structures A, B, and C in Figure 3d, with the n-type region represented by symbol 1, the MQWs represented by symbol 2, the EBL-I represented by symbol 3, the EFR represented by symbol 4, the EBL-II represented by symbol 5, and the p-type region represented by symbol 6. It can be seen that the electric field distribution is basically the same in the n-type region and the active region. However, in EBL-I, the three structure holes are subjected to an electric field E1 with an electric field in the direction of [0001], and the direction of the electric field force on the hole is opposite to the direction of the hole’s motion, but in the EFR, structure C appears with electric field E2 in the direction of [000-1]. In EBL-II, structures B and C are subjected to electric field E3 in the direction of [000-1], and the direction of the electric field force on the hole in EBL-II is consistent with the direction of the motion of the hole. The rate of the transport of the hole depends on the average strength (E) and direction (±) of the electric field, i.e., the work (W) conducted by the electric field on the hole. The expression for the work conducted by the electric field on the hole is shown in Equation (4) as:

(4) $$W=\int q\cdot d\cdot ed_{E},$$

where *q* is the electron charge, *d* is the distance traveled by the hole, and *e* is the average intensity.

In structure A, the work conducted by the electric field on the electrons is W ∝ (−E1), in structure B, the work conducted by the electric field on the electrons is W ∝ (−E1 + E3), and in structure C, the work conducted by the electric field on the electrons is W ∝ (−E1 + E2 + E3), and the work conducted by the electric field for all three structures is in the following order: structure C > structure B > structure A. For structure C with the EFR, the electric field does the most work on the holes, and the holes are transported fastest in the p-EBL.

**Figure 4:**
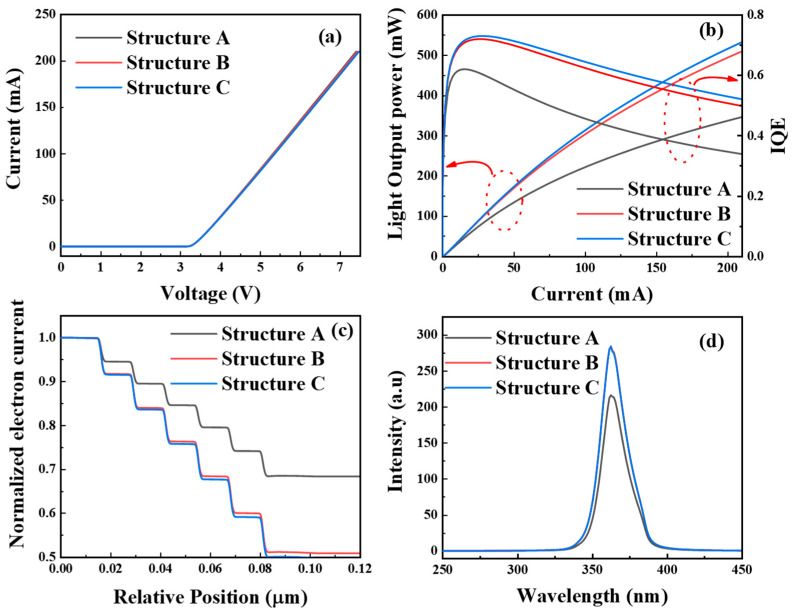
(**a**) I-V and electro-luminescence spectra at 210 mA, (**b**) calculated optical power and IQE, (**c**) normalized electron current at 210 mA, (**d**) EL spectrum at room temperature.

The I-V diagrams of structures A, B, and C are given as shown in Figure 4a. The results show that the device can conduct when the applied voltage is about 3.2 V, and the on-state current is about 1 μA and structures B and C need slightly higher voltage values to achieve the same current value compared to structure A. As shown in Figure 4b, the light output power plots and IQE plots are given for structures A, B, and C, respectively. The slope of the light output power curves of structures B and C is greater, and the light output power increases significantly. When the current is 100 mA, the light output power of structure A is 224 mW, and the light output power of structure B is 308 mW, which is about 84 mW different from structure A. The light output power of structure C is 318 mW, which is about 94 mW different from structure A. The light output power of structure C is 318 mW, and the light output power of structure A is about 94 mW different from structure A. The light output power of structure B is 308 mW, and the light output power of structure C is about 84 mW. When the current is increased to 210 mA, the light output power of structure A is 346 mW, and the light output power of structure B is 509 mW, which is about 163mW different from that of structure A. The light output power of structure C is 532 mW, which is about 186 mW different from that of structure A. The light output power is an important index to characterize the optoelectronic performance of LEDs, and therefore, the light output power of the polarized EBL-LED model has a great advantage compared with that of the traditional EBL-LED. Therefore, the light output power of the polarized EBL-LED model structure has a great advantage compared with that of the traditional EBL-LED, which proves that the polarized EBL-LED model can indeed improve the light-emitting ability of AlGaN/InGaN UV LEDs. And the EFR does further improve the luminescence ability of UV LEDs. Structures B and C have higher IQE under the condition of increasing the overall current, and the sag value is defined as (IQE max − IQE 210 mA)/IQE max × 100%, which indicates the degree of efficiency sag at a high injection current. The efficiency drop of structures B and C is significantly suppressed. Structure A decreases from 61.9% to 33.9% with a sag value of 45%, while structure B decreases from 72% to 49.8% with a sag value of 30.8%. Structure C decreased from 73% to 52.1% with a sag value of 28.6%. This indicates that the polarized EBL with an EFR layer has better electron blocking ability and promotes hole injection. The IQE increases and then decreases when the current is continuously increased. This is because the carrier concentration is very high when the current is continuously increased, and the main reason for the decrease in the luminous efficiency of the UV LED structure changes from electron leakage to an increase in the nonradiative compounding, which is unavoidable for all three structures.

In order to reveal the electron leakage problem that causes efficiency degradation in UV-LEDs, we plotted the normalized electron currents in the p-type layer in Figure 4c, where a significant reduction in electron leakage from the active region can be observed. About 70% of the electron current in structure A leaks into the p-type layer, whereas only 50% of the electrons leak out of structure B compared to A. Due to the improvement in the electron blocking ability and hole injection efficiency, the output performance of structures B and C is enhanced, so the optical output power and IQE of structures B and C are improved, and the optical output power and IQE are further improved by the thermionic emission and tunnelling effect induced by the local electric field of the EFR in structure C. This indicates that the LEDs using polarized EBL structures with EFR layers have a significant blocking effect on the electron leakage and promote hole injection to some extent. As shown in Figure 4d, the EL spectra of the three structures at room temperature are given, and the calculated EL spectra show that structures A, B, and C have the same peak emission wavelength at an injection current of 210 mA. It is also observed that the EL intensity of structures B and C is increased by more than 0.3 times compared to structure A.

## 4. Discussion

The use of a polarized EBL and the introduction of the EFR are beneficial to improve the IQE and optical output power of UV LEDs, mainly because polarized EBLs reduce the valence band barrier and improve the conduction band barrier by using the polarization effect, whereas the EFR promotes hole injection by using the built-in local electric field to improve the efficiency of hole injection and improve the hole drift rate due to the reduction in the valence band, which greatly improves the hole tunneling probability. The simulation results of the polarized EBL structure with the EFR show that due to the introduction of the EFR, the electron barrier is enhanced, and its electron blocking ability is significantly strengthened, and with the introduction of a local electric field in the EFR layer, the hot-ion emission and tunnelling effects are enhanced, and the hole injection ability is greatly improved. This structure is favorable to reduce the electron leakage in the active region as well as to improve the hole injection difficulty in the p-type region. The section on the effect of thickness on LED photovoltaic performance has been re-added as follows: too high a thickness of the EFR layer results in too high a dislocation density, which leads to a decrease in the internal quantum efficiency; too low a thickness of the EFR layer results in too low a strength of the energy storage electric field, and there are not enough holes to pass through the potential barriers.

On this basis, compared to other UVLEDs, and compared to Wang [34,35], with the same wavelength of light emission, the light output power is greatly improved, and the growth structure is simpler. This ensures a high optical output power at reduced wavelengths compared to Yue [36].

## 5. Conclusions

In this work, UV LEDs with a peak value of 365 nm were systematically investigated using the APSYS (2016 version) commercial software to study the energy band changes due to the polarization effect, as well as the effect of the localized electric field with the EFR on hole injection, and the results showed a significant increase in the optical output power by a factor of 0.53 and an increase in the IQE by 19% compared to the conventional UV-LEDs. The results show that polarized EBLs with the EFR have a great potential in high-efficiency UV-LEDs. And the UV-LED structure and device physical characteristics proposed in this paper are favorable for the development of UV-LEDs.

## Figures and Tables

**Table 1:** Electron barriers (Φe) and hole barriers (Φh) in three structures.

	EBL-I	EBL-II
Structure A	Φ1e = 206.25 Φ1h = 286.44	
Structure B	Φ2e = 262.91 Φ2h = 290.86	Φ3e = 262.18 Φ3h = 123.56
Structure C	Φ4e = 209.18 Φ4h = 272.66	Φ5e = 207.89 Φ5h = 233.03

## Data Availability

All data that support the findings of this study are included within the article.
